# Electrochemical Determination of Fentanyl Using Carbon Nanofiber-Modified Electrodes

**DOI:** 10.1021/acsomega.4c00816

**Published:** 2024-04-05

**Authors:** Armando J. Marenco, Rajesh G. Pillai, Kenneth D. Harris, Nora W. C. Chan, Abebaw B. Jemere

**Affiliations:** † National Research Council Canada—Nanotechnology Research Centre, 11421 Saskatchewan Drive, Edmonton, Alberta T6G 2M9, Canada; ‡ Department of Mechanical Engineering, University of Alberta, Edmonton, Alberta T6G 1H9, Canada; § Defence Research and Development Canada, Suffield Research Centre, P.O. Box 4000, Stn. Main, Medicine Hat, Alberta T1A 8K6, Canada; ∥ Department of Chemistry, Queen’s University, Kingston, Ontario K7L 3N6, Canada

## Abstract

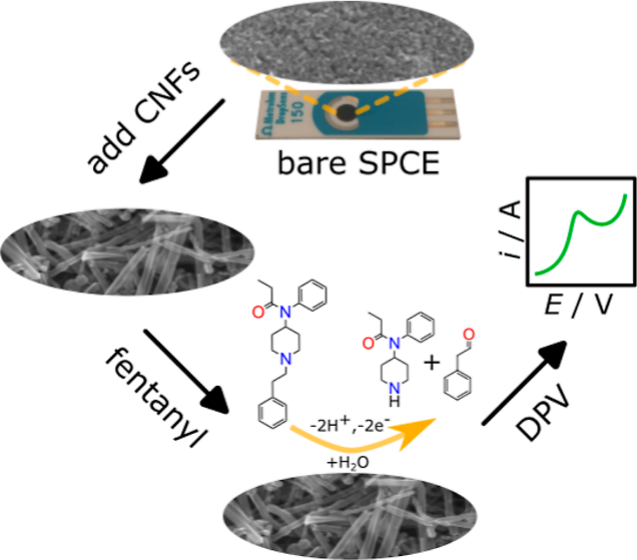

In this work, we report the direct electrochemical oxidation of fentanyl using commercial screen-printed carbon electrodes (SPCEs) modified with carboxyl-functionalized carbon nanofibers (fCNFs). CNFs have surface chemistry and reactivity similar to carbon nanotubes (CNTs), yet they are easier to produce and are of a lower cost than CNTs. By monitoring the current produced during the electrochemical oxidation of fentanyl, variables such as fCNF loading, fentanyl accumulation time, electrolyte pH, and differential pulse voltammetry parameters were optimized. Under an optimized set of conditions, the fCNF/SPCEs responded linearly to fentanyl in the concentration range of 0.125–10 μM, with a limit of detection of 75 nM. The fCNF/SPCEs also demonstrated excellent selectivity against common cutting agents found in illicit drugs (e.g., glucose, sucrose, caffeine, acetaminophen, and theophylline) and interferents found in biological samples (e.g., ascorbic acid, NaCl, urea, creatinine, and uric acid). The performance of the sensor was also successfully tested using fentanyl spiked into an artificial urine sample. The straightforward electrode assembly process, low cost, ease of use, and rapid response make the fCNF/SPCEs prime candidates for the detection of fentanyl in both physiological samples and street drugs.

## Introduction

The opioid epidemic is a great concern worldwide. According to the United Nations Office on Drugs and Crime, approximately 90,000 lives were lost in 2019 due to opioid overdoses worldwide and in 2021, approximately 88,000 lives were lost in North America alone.^1^ Among the synthetic opioids, fentanyl {*N*-phenyl-*N*-[1-(2-phenylethyl)-4-piperidinyl]-propanamide} is one of the most useful and potent opioids in medicinal pain management;^2^ however, it has become widely misused and abused. When fentanyl is therapeutically administered, significant analgesia may occur at plasma concentrations as low as 0.6–3.6 nM, with the effect occurring as soon as 1–2 min after injection and lasting for 2–4 h.^3^ In blood, the lethal dose associated with fentanyl use shows tremendous variation, ranging in various studies from 3 to 600 nM.^4−6^ Though a number of laboratory techniques including surface-enhanced Raman spectroscopy,^7^ enzyme-linked immunosorbent assays,^8^ high-performance liquid chromatography coupled with UV spectroscopy readout,^9,10^ gas chromatography–mass spectrometry,^11^ capillary-based immunosensing,^12^ and ion mobility mass spectrometry^13^ have been shown to have promising detection capabilities toward fentanyl, liquid chromatography–mass spectrometry remains the “gold standard” for fentanyl determination.^14−16^ These techniques, although sensitive, are also costly, requiring elaborate protocols, highly trained personnel, and large, complex pieces of equipment, and thus, they tend to be restricted to centralized laboratory settings. In-field testing, however, would be incredibly beneficial in attempts to combat the opioid epidemic: first responders, for example, would gain a useful tool to diagnose unresponsive patients, and contaminants could be identified in drug supplies before consumption, preventing deaths or new addictions. In these applications, fast, inexpensive, and portable sensors with high selectivity and low limits of detection (LODs) are essential. Electroanalytical methods exhibit such advantages^17,18^ and are prime candidates for the development of fentanyl sensors.

In recent years, a number of reports have described electrochemical measurements of fentanyl, the majority of which are based on carbon electrodes such as carbon nano-onions,^19^ carbon nanotubes (CNTs),^20−22^ carbon paste microneedles,^23^ screen-printed carbon electrodes (SPCEs),^24^ laser-ablated porous carbon electrodes,^25^ and graphene oxide-modified electrodes.^26^ Recent review articles on electrochemical sensors for fentanyl detection underscored the need for not only higher-performance devices capable of detecting extremely low concentrations (<80 nM) of fentanyl in both street drugs and in biologically relevant solutions but also the development of low-cost disposable sensors.^27,28^ The above-cited literature establishes that the oxidation of the drug is adsorption-driven,^19−26^ which can potentially create fouling effects from byproducts and other interferents present in fentanyl-containing samples. When this cross-contamination is an expected issue, the use of low-cost, disposable materials in sensor fabrication is advantageous.

In this study, we therefore utilized carboxyl-functionalized carbon nanofiber (fCNF)-modified SPCEs for the direct electrochemical detection of fentanyl. To the best of our knowledge, this is the first demonstration of selective and sensitive electrochemical determination of fentanyl using commercially available CNFs. CNFs have surface chemistry and reactivity similar to CNTs, yet they are easier to produce and of a lower cost than CNTs. The overall reproducibility of the sensor (fCNF/SPCE) fabrication and performance was high [relative standard deviations (% RSD) of <5% within a single batch, *N* = 8, and <10% for batch-to-batch variability, *N* = 3]. The developed sensor also displayed excellent storage stability for at least 8 weeks. Furthermore, the sensor was able to discriminate fentanyl in the presence of common fillers or cutting agents found in street drugs, and it was not susceptible to interference from components of human urine, responding linearly for 1–10 μM fentanyl spiked into artificial urine samples.

## Experimental Section

### Reagents and Materials

Cerilliant-certified reference solutions of fentanyl and norfentanyl (1.0 mg mL^–1^ in methanol), conical CNFs, sodium phosphate dibasic, sodium phosphate monobasic, sulfuric acid, nitric acid, ethanol, potassium ferrocyanide, potassium ferricyanide, potassium nitrate, ascorbic acid, urea, glucose, sucrose, NaCl, caffeine, creatinine, uric acid, acetaminophen, theophylline, and phenylacetaldehyde were purchased from Sigma-Aldrich Canada (Oakville, ON). Artificial urine was purchased from Pickering Laboratories (Mountain View, CA, USA). All chemicals were used without further purification. SPCEs consisting of a working C electrode (4 mm in diameter), a Pt auxiliary electrode, and a Ag reference electrode were acquired from Metrohm DropSens (DPR-150, Oviedo, Spain). Deionized (DI) water with 18 MΩ·cm resistivity (Millipore Canada, Mississauga, ON) was used throughout this work for solution preparation and electrode rinsing. Fentanyl and norfentanyl stock solutions were prepared by evaporating the bulk methanol solvent overnight at room temperature using a gentle N_2_ stream in a fumehood, followed by a 30 min high-vacuum drying process to further remove trace solvent. Complete removal of methanol was necessary as we observed that it undergoes oxidation at a potential similar to fentanyl. Separate stock solutions of fentanyl (0.594 mM) and norfentanyl (0.620 mM) were then prepared by resuspending each chemical in DI water. Daily working solutions were prepared in 0.1 M phosphate buffer (PB), pH 8.0, except during the pH optimization study.

### fCNF/SPCE Sensor Fabrication

First, acid functionalization of CNFs was conducted as per a published protocol.^29^ Briefly, 1 g of the as-received CNF was added to 40 mL of HNO_3_:H_2_SO_4_ (1:3, v/v), followed by stirring for 8 h at 50 °C. The suspension was then washed with 150 mL of DI water and centrifuged. The resulting pellet was resuspended with DI water, and the washing process was repeated multiple times until a pH of ∼6 was obtained. The fCNFs were freeze-dried and stored under a N_2_ environment before use. Raman spectra (DXR2, Thermo Scientific, Toronto, ON, Canada) and zeta potentials (Zetasizer, Malvern Panalytical, St. Laurent, QC, Canada) of pristine and functionalized CNFs were measured and compared. Working solutions were prepared by transferring 0.25–3 mg of fCNFs to 1 mL of ethanol and resuspending by sonicating for 25 min to disperse the fCNFs.

**Scheme 1:**
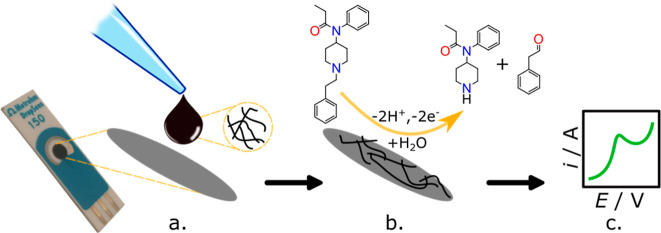
fCNF/SPCE Fabrication and Testing Process Consisting of (a) Drop-Casting fCNFs onto the SPCE, (b) Incubation and Electrochemical Oxidation of Fentanyl, and (c) Data Collection and Analysis

The fCNF/SPCE sensor construction was carried out by a simple one-step process, as shown in Scheme 1. Prior to modifying with fCNFs, a bare SPCE was electrochemically cleaned by cycling in 0.1 M H_2_SO_4_ between −0.2 and 1.3 V vs Ag at 100 mV s^–1^. Stable cyclic voltammograms (CVs) were typically obtained after 5 cycles. Then, 8 μL of fCNF ethanolic suspension was drop-cast onto the C working electrode and allowed to dry at room temperature for a minimum of 30 min, leaving a thin film of dry fCNFs. The fCNF/SPCE was then rinsed with DI water, dried with a gentle stream of N_2_, and electrochemically cleaned under the same conditions as the bare SPCE immediately prior to testing. Unused fCNF/SPCEs were stored under vacuum in a desiccator.

### Electrochemical Measurements and Data Analysis

All electrochemical measurements were performed using a Reference 600 Gamry potentiostat controlled by Framework data acquisition software, version 7.10.0 (Gamry Instruments Inc., Warminster, PA). To conduct electrochemical measurements, 150 μL of the solution was pipetted onto the electrode surface. The electrochemically active surface areas (ECSAs) of the bare SPCE and fCNF/SPCE were determined by recording CVs of 1 mM K_3_[Fe(CN)_6_]/K_4_[Fe(CN)_6_] in 0.1 M KNO_3_ solution at different scan rates. Plots of the anodic peak currents vs square roots of scan rate were linearly fitted, and the slope of the fits was utilized to determine ECSA according to the Randles–Sevcik equation.^30^ Differential pulse voltammetry (DPV) measurements were carried out by scanning the potential from +0.4 to 1.2 V (unless otherwise specified) at room temperature, using a pulse amplitude of 100 mV, scan rate of 100 mV s^–1^, and pulse period of 20 ms. Electrochemical impedance spectroscopy (EIS) was performed in a frequency range of 0.1 Hz to 100 kHz using an AC amplitude of 10 mV at open-circuit potential. Before quantitative electrochemical measurements of target analytes, the electrodes were passively incubated with the analytes (i.e., without stirring or applied potential), and the duration of the incubation is termed accumulation time.

Data treatment for the ternary mixture consisting of fentanyl, acetaminophen, and theophylline was performed with OriginPro software version 10.05. DPV voltammograms were analyzed by using the peak analyzer function. Baseline subtraction was applied by using the spline method. Peak deconvolution of the DPV ternary mixture voltammogram consisted of multiple peak function analyses using a Gaussian peak type. Morphology of the modified electrodes was characterized by scanning electron microscopy (Hitachi S-4800, Japan).

## Results and Discussion

### Characterization of the fCNF-Modified Electrode

Scheme 1 shows the steps followed in the fabrication of the fCNF/SPCE fentanyl sensor. First, the SPCE was modified with carboxylic acid-functionalized CNFs to increase the electrical conductivity and specific surface area of the electrode. The functionalization of CNFs with −COOH groups was characterized by Raman spectroscopy and zeta potential measurements. As shown in Supporting Information Figure S1a, the Raman spectra of both pristine and functionalized CNFs exhibit three major peaks centered at ∼1350, ∼1590, and ∼2690 cm^–1^ which correspond to the D, G, and 2D bands of CNFs, respectively.^31^ The intensity ratio of the D and G peaks, which is usually used to characterize structural defects in graphitic structures, increased from 0.1 for CNFs to 0.4 for fCNFs, implying that the acid functionalization increased the disorder.^31^ The zeta potentials of unfunctionalized and functionalized CNFs, measured in water, were found to be −24.1 ± 2.8 and −45.3 ± 0.6 mV, respectively. The more negative value measured for fCNFs indicates the presence of additional negative charges, which are imputed to the introduction of −COOH functional groups.

The electrochemical properties of the bare SPCE and fCNF/SPCE were characterized by recording CVs of 1 mM [Fe(CN)_6_]^3–/4–^ in 0.1 M KNO_3_ as shown in Supporting Information Figure S1b. As expected, the CV of the bare SPCE shows a quasi-reversible redox behavior with a peak-to-peak separation potential (Δ*E*_p_) of 329 mV at 100 mV s^–1^. Modification of SPCEs with gradually increased amounts of fCNFs led to a decrease in Δ*E*_p_ while displaying quasi-reversible redox behavior. For example, modification with the lowest-concentration fCNF suspension of 0.25 mg mL^–1^ reduced the Δ*E*_p_ value to 264 mV, while addition of the 3 mg mL^–1^ fCNF suspension resulted in a Δ*E*_p_ of 99 mV. This trend indicates that fCNFs promote electron transfer between the redox probe and the SPCE. This observation is also supported by EIS measurements of the same electrodes as shown in Figure S1c. The charge-transfer resistance (*R*_CT_) values obtained by fitting the Bode plots with the Randles circuit (inset in Figure S1c) indicate a drastic increase in conductivity with the addition of fCNFs to the SPCEs. The initial *R*_CT_ of an unmodified SPCE is ∼12 kΩ, while the addition of the 0.25 mg mL^–1^ fCNF suspension lowered this value to ∼7.5 kΩ. Modifications with fCNF suspensions ranging from 0.5 to 3 mg mL^–1^ resulted in *R*_CT_ values of <1 Ω. From CVs of the various electrodes tested at different scan rates (10–400 mV s^–1^) in the presence of 1 mM [Fe(CN)_6_]^3–/4–^, plots of peak currents vs square roots of scan rate are presented in Figure S1d. The corresponding ECSAs of the electrodes, calculated using the Randles–Sevcik equation,^30^ are also shown in Supporting Information Figure S1d. It is evident that the fCNF-modified electrodes have higher ECSA values, and these ECSAs increase with the amount of fCNF used in the modification (inset table in Figure S1d). However, we also observed that the stability and integrity of the fCNF/SPCEs degraded as the amount of fCNF was increased beyond 1 mg mL^–1^. As discussed below in Section 3.3, the 1 mg mL^–1^ fCNF/SPCE sensor also yielded the highest fentanyl oxidation current; thus, SPCEs modified with 1 mg mL^–1^ fCNFs were selected for subsequent studies. SEM images of a 1 mg mL^–1^ fCNF-modified electrode are shown in Supporting Information Figure S1e, where the presence of fCNFs was evident following modification.

### Cyclic Voltammetry of Fentanyl Using the fCNF/SPCE

**Figure 1:**
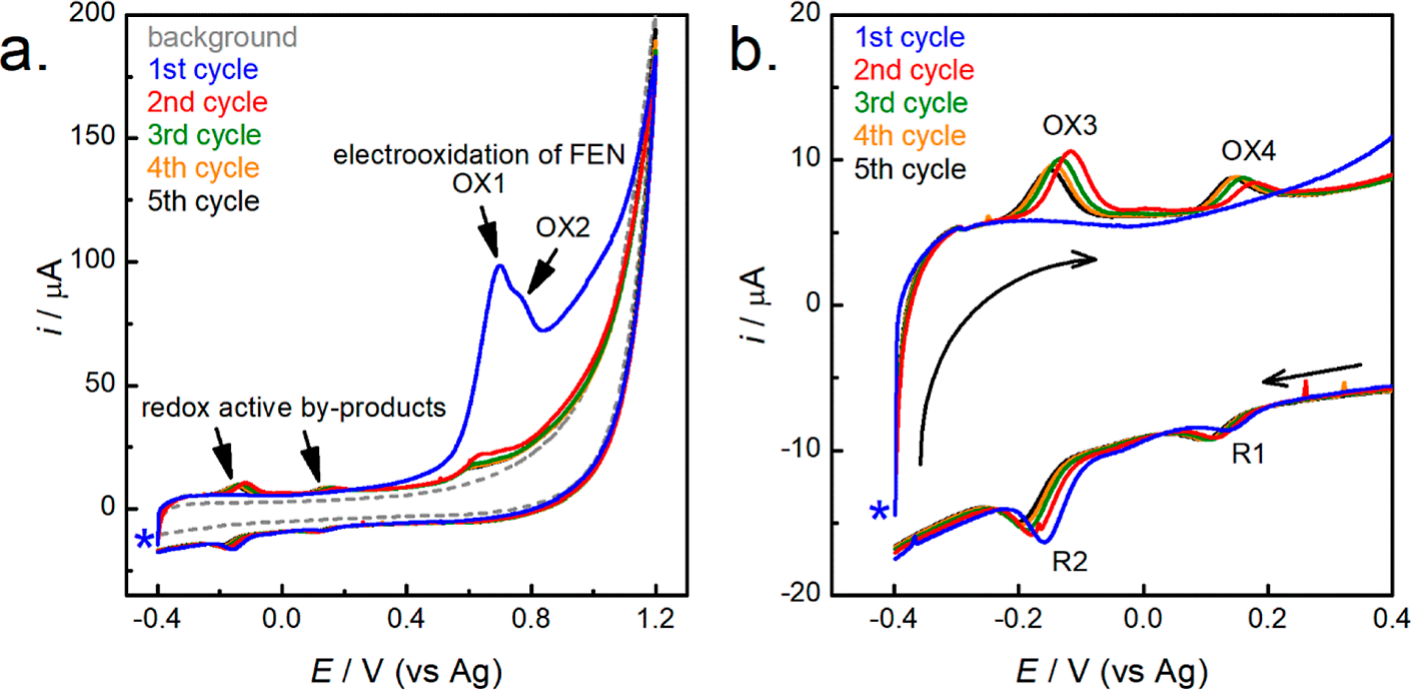
First five consecutive CVs for an SPCE modified with a 1 mg mL^–1^ fCNF suspension in the absence (gray dashed line) and presence (solid lines) of 75 μM fentanyl. Oxidation peaks have been labeled with OX and reduction peaks with R in the order of appearance during CV recording. The potential window ranging from −0.4 to 1.2 V is shown in (a), while (b) is a close-up of the region from −0.4 to +0/4 V showing redox activity of the electrogenerated byproducts. In (a) and (b), the blue asterisk indicates the start of the first scan while in (b), the black arrows indicate the direction of the scans. Testing was conducted under unoptimized conditions at 100 mV s^–1^ in 0.1 M PB buffer (pH 8), and the electrode was incubated in fentanyl for 3 min prior to testing.

Figure 1 shows successive CVs of a modified fCNF/SPCE in the presence and absence of fentanyl. In the absence of fentanyl, the fCNF/SPCE shows no observable peaks (gray dashed line in Figure 1a), while the same electrode exhibited multiple peaks in the presence of 75 μM fentanyl, resulting from the electrochemical oxidation of the drug as previously reported for carbon, Zn(II)-MOF, and ionic-liquid electrodes.^19−21,24,25,32,33^ During the first cycle (blue trace in Figure 1), the forward anodic sweep shows two peaks (labeled OX1 and OX2) at 0.70 and 0.77 V. During subsequent scans, limited time is provided for fentanyl to reaccumulate on the electrode, and thus the intensities of OX1 and OX2 are much reduced indicating consumption of fentanyl during electro-oxidation. The oxidation of fentanyl on the fCNF/SPCE is irreversible as corroborated by the lack of related cathodic peaks in subsequent reverse scans. In the literature, the electrochemical oxidation of fentanyl is proposed to proceed through a two-electron-coupled two-proton dealkylation of its piperidine nitrogen (a tertiary amine) followed by a hydrolysis step ultimately resulting in norfentanyl (a secondary amine) and phenylacetaldehyde.^19−21,24,25,32,33^ The reverse cathodic sweep of the first cycle shows two small peaks at 0.12 and −0.18 V, labeled R1 and R2, respectively, resulting from the reduction of the byproducts of fentanyl oxidation. The second cycle (red trace in Figure 1) reveals two new anodic peaks (OX3 at −0.12 V and OX4 at 0.18 V) that are not observed during the first cycle. Figure 1b depicts a close-up of a region of the CV presented in Figure 1a showing the redox activity of the electrogenerated byproducts. The redox pair OX3 and R2 is ascribed to the electrochemical redox behavior of norfentanyl on the fCNF/SPCE surface as has been established by others.^19,20,26^ (We also conducted an independent CV experiment using our fCNF/SPCE in norfentanyl solution and observed redox pairs at the same potentials; see Supporting Information Figure S2a,b). Phenylacetaldehyde, on the other hand, is not electroactive on the fCNF/SPCE (Supporting Information Figures S2c,d), and to the best of our knowledge, no literature report shows that it can be electrochemically oxidized or reduced. Thus, the source of R1 and OX4 observed in our sensor is currently unknown but was reproducibly observed for the different fCNF-modified SPCEs as presented in Supporting Information Figure S3.

In the literature, some authors argue that the two oxidation peaks, OX1 and OX2 observed at sufficiently high scan rates, originate from the oxidations of the tertiary amine of fentanyl (OX1) and the newly formed secondary amine, norfentanyl (OX2).^17,18,23^ The latter assignment, however, is not supported by our data in Figure S2a,b, as norfentanyl did not show peaks at similar potentials. Similar observations of the lack of norfentanyl oxidation peaks at high potential have also been reported by both Ott et al.^24^ and Goodchild et al.^33^ Ott et al. hypothesized that both peaks originate from oxidation of the same tertiary amine, with OX1 and OX2 originating from fentanyl adsorbed at and diffusing to the sensor surface, respectively.^24^ Moreover, the theoretical and experimental work of Compton’s group on the CV of redox chemicals at CNT-modified electrodes shows that analyte adsorption and thin-layer diffusion can contribute to two distinct oxidation peaks, with the adsorbed species oxidizing at a lower potential than the diffusing species.^34,35^ Given this information and our observed lack of oxidation peaks for both norfentanyl and phenylacetaldehyde at potentials similar to those of OX1 and OX2, we believe that our data tend to support Ott’s hypothesis, and we assign OX1 and OX2 to the oxidation of adsorbed and diffusing fentanyl, respectively.

**Figure 2:**
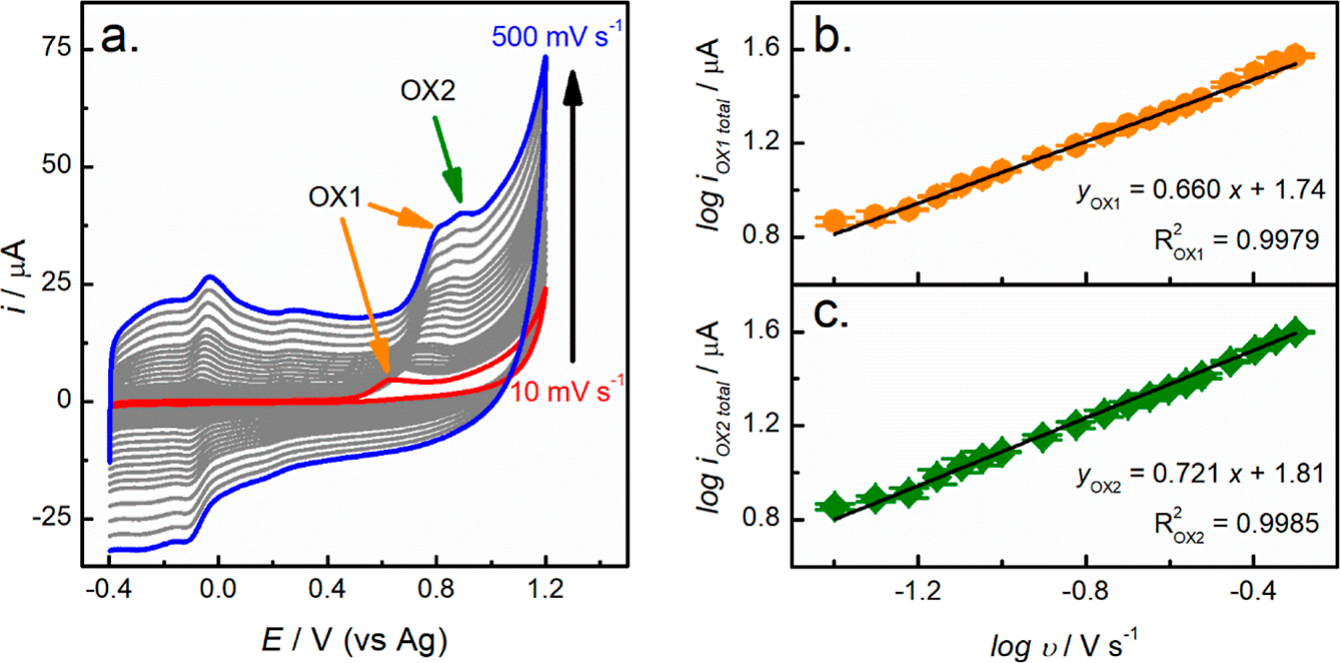
Cyclic voltammetry of the 1 mg mL^–1^ fCNF/SPCE in the presence of 75 μM fentanyl in 0.1 M PB pH 8. (a) Effect of scan rates on the CV, and (b) log of peak currents for OX1 and OX2 as a function of log of scan rate (*N* = 2 electrodes).

In order to further understand the oxidation of fentanyl by fCNF/SPCEs, we conducted cyclic voltammetry of fentanyl under varying scan rates (10–500 mV s^–1^), as shown in Figure 2. From Figure 2a, it is evident that at scan rates greater than 40 mV s^–1^, the oxidation of fentanyl gives two oxidation peaks (OX1 and OX2) whose peak potential separation (Δ*E*_p_) remains relatively constant at 72 ± 8 mV. (A similar value of 88 ± 7 mV at scan rates above 100 mV s^–1^ has been reported using glass-supported SWCNTs^20^). At scan rates below 30 mV s^–1^, however, where the diffusion layer is relatively large and transport of fentanyl from solution to the electrode is slower, only a single peak appears. Plotting the logarithm of the oxidation peak currents vs the logarithm of scan rate, Figures 2b,c, yielded linear relationships with slopes of 0.66 (*R*^2^ = 0.9979) and 0.72 (*R*^2^ = 0.9985) for OX1 and OX2, respectively, indicating that the oxidation of fentanyl is governed by both diffusion and adsorption processes. Similar electrochemical processes have been reported by others for the electrochemical oxidation of fentanyl on carbon-based and Zn-MOF electrodes.^19,21,24,32^ The significance of the adsorption of fentanyl onto the fCNF/SPCE surface is also supported by a study of the influence of fentanyl incubation (adsorption) time on the intensity of the oxidation peak, discussed below.

### Optimization of the fCNF/SPCE Sensor Fabrication

**Figure 3:**
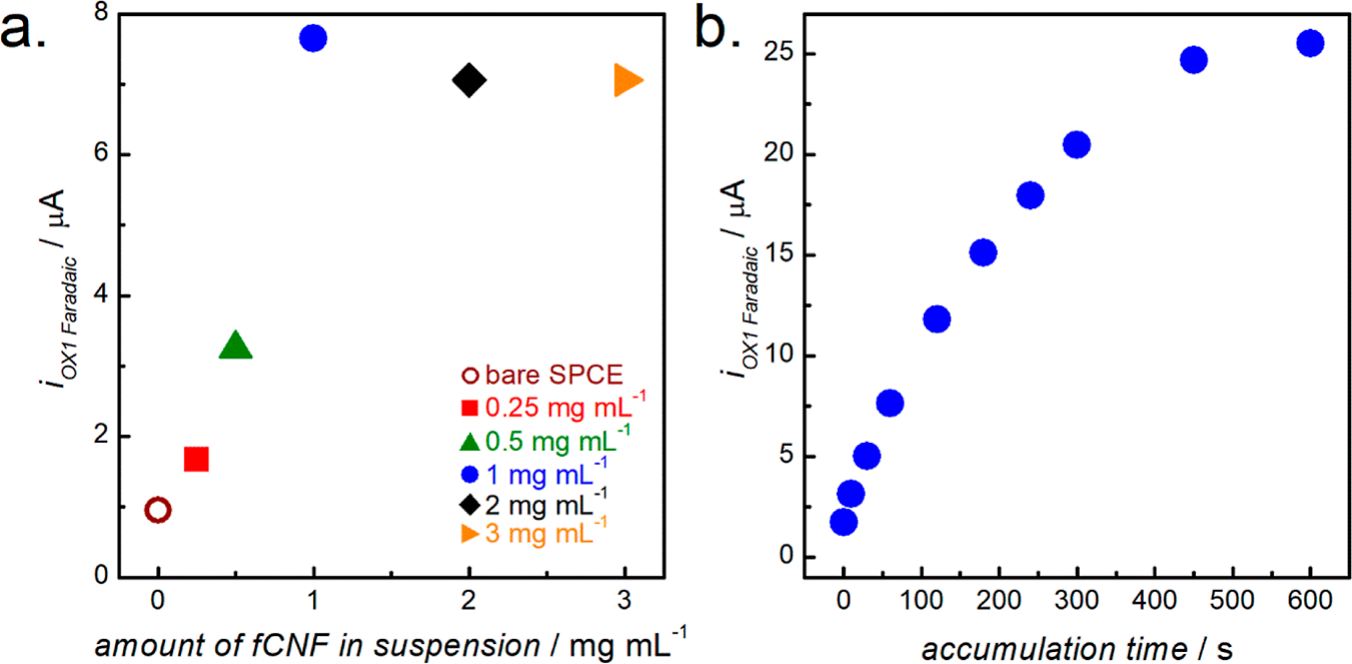
Current responses resulting from the electro-oxidation of 75 μM fentanyl using a bare SPCE and SPCEs modified with different fCNF suspensions. (a) Oxidation current response showing the effect of fCNF loading on the SPCE after incubation for 60 s in fentanyl. (b) Oxidation current from a 1 mg mL^–1^ fCNF/SPCE exposed to fentanyl for different lengths of time prior to CV measurement. Testing was conducted in 0.1 M PB buffer at pH 8.0 and 50 mV s^–1^

Parameters affecting the sensor performance, including the amount of fCNF in the modification solution, fentanyl accumulation time, and electrolyte pH, were optimized. Supporting Information Figure S4 shows a CV comparison of a bare SPCE and several fCNF/SPCEs after being incubated with 75 μM fentanyl for 60 s. Fentanyl shows an irreversible oxidation peak for both bare SPCE and modified electrodes, but fCNF-modified electrodes exhibited oxidation at lower potentials and higher peak currents than the bare electrode, demonstrating improved charge transfer and catalytic properties imparted by fCNFs. As shown in Figure 3a, a plot of the OX1 peak current as a function of fCNF loading exhibits a maximum at 1 mg mL^–1^ fCNFs. Further increasing the fCNF loading did not yield an increased fentanyl oxidation current but caused sensor instability and performance fluctuations (as discussed in Section 3.1, thicker films flaked off during rinsing and measurements), contributing to the slightly lower fentanyl oxidation currents shown in the figure.

The observed fentanyl oxidation peak current is directly related to the amount of analyte in the vicinity of the electrode. Therefore, the effect of the drug’s accumulation time on the oxidation current of fentanyl was investigated, with results for the 1 mg mL^–1^ fCNF/SPCE shown in Figure 3b. The figure reveals that the fentanyl oxidation current initially increased with accumulation time before reaching a plateau at about 450 s of accumulation, indicating sensor saturation. This time was selected as the optimal accumulation time, and it is roughly comparable to the 300–500 s previously reported for carbon nano-onions,^19^ CNTs,^20,21^ and SPCEs.^24^

**Figure 4:**
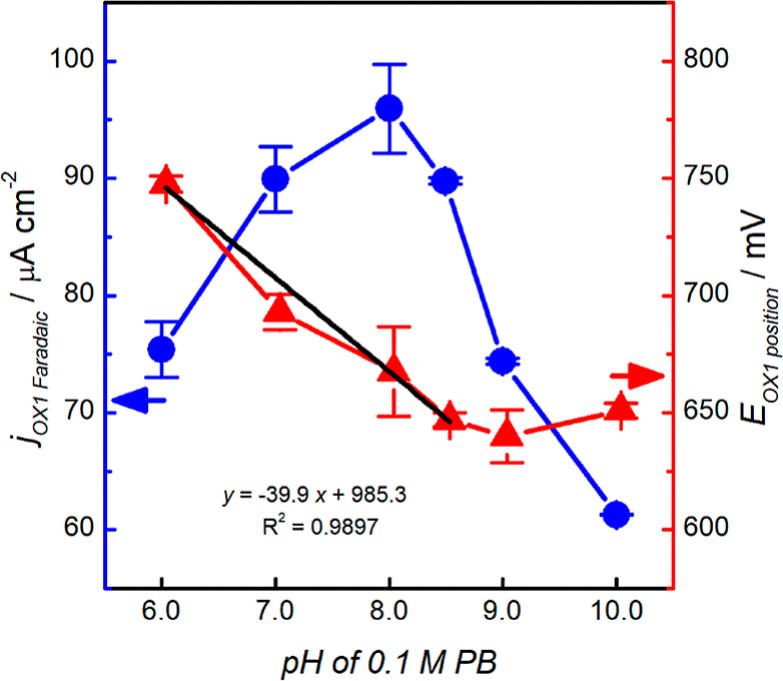
Effect of pH on the fentanyl OX1 peak current density (blue, left *y*-axis) and peak position (red, right *y*-axis) in 0.1 M PB buffer during CV measurements (*N* = 3 electrodes). The black straight line is a linear fit of a portion of the *E*_ox1_ vs pH data.

The pH of the electrolyte solution also affects the electrochemical determination of an analyte due to the charge on the electrode surface and the dissociation of the analyte. Here, we studied the effect of pH on the fentanyl oxidation potential and peak current using the CV technique. As shown in Figure 4, the maximum current was obtained at pH 8.0, which was used in subsequent measurements. Figure 4 also shows that the oxidation potential of fentanyl increases as the electrolyte pH is reduced, indicating that protons are involved in the oxidation process. The plot of peak potential vs pH yielded a straight line between the pH values of 6.0 and 8.5 with a slope of 40 mV pH^–1^ unit, which is close to the theoretical Nernstian value of 59 mV pH^–1^ unit predicted for an equal number of protons and electrons involved in an electrochemical reaction. The deviation (equivalent to a proton/electron ratio of 0.7 rather than the ideal value of 1) is ascribed to the different protonation states of fentanyl in these pH ranges. At pH values above 8.5, the oxidation potential is no longer proton-coupled, suggesting complete deprotonation of the drug with a p*K*_a_ slightly above 8. (Reported p*K*_a_ of fentanyl is 8.44 at 25 °C^27^).

### Analytical Performance of fCNF/SPCE Sensors

**Figure 5:**
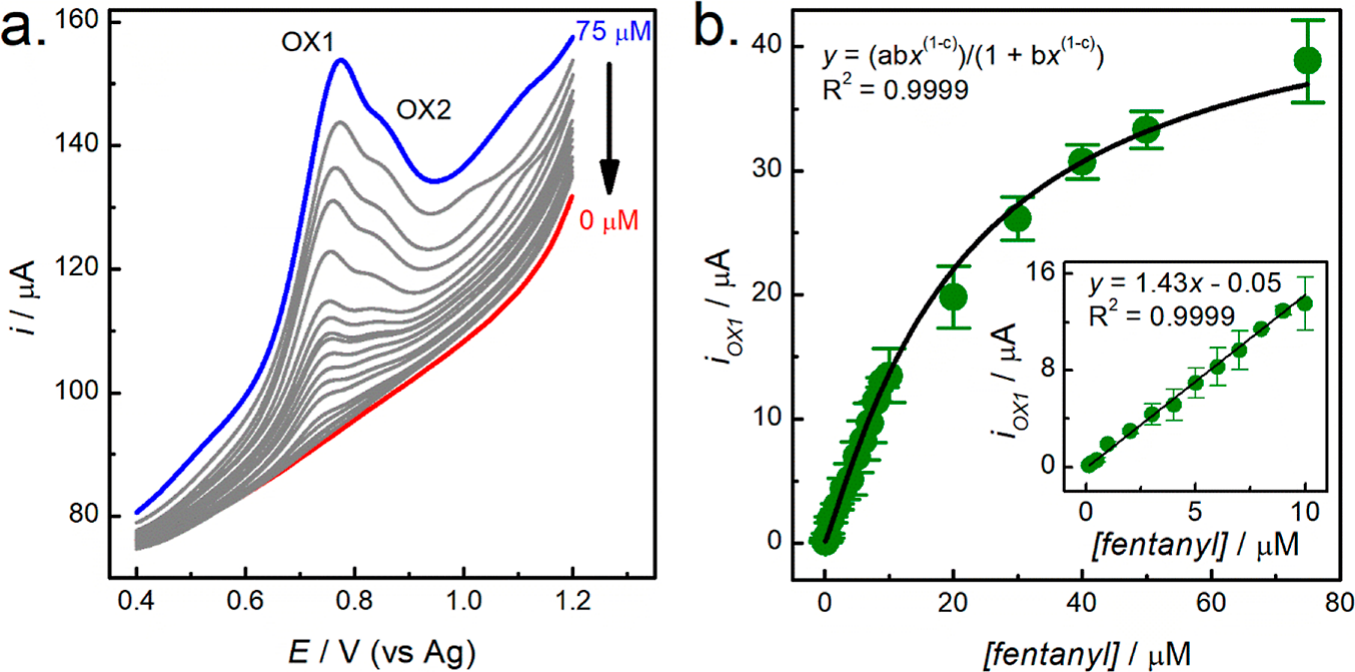
(a) DPV voltammograms for a 1 mg mL^–1^ fCNF/SPCE in different fentanyl concentrations and (b) extracted current measurements from 3 electrodes with a Langmuir fit (black trace). The fitted Langmuir equation constants are *a* = 46 μA, *b* = 32 × 10^–3^ μM^–1^, and *c* = −120 × 10^–3^. The inset in (b) is the zoomed-in region of fentanyl concentrations ≤10 μM with its independent linear fit.

The analytical performance of the fCNF/SPCE sensor for the electrochemical determination of fentanyl was evaluated using optimized DPV parameters (optimization data shown in Figure S5). Figure 5a shows representative DPV responses for fCNF/SPCE sensors to increasing concentrations of fentanyl. In general, the DPV responses have features similar to the CVs; in particular, the OX1 peak is accompanied by an OX2 shoulder. The plot of OX1 peak current vs the concentration of fentanyl is shown in Figure 5b, where each data point was obtained from three different batches of electrodes, with the error bars showing standard deviations of the measurements. The batch-to-batch sensor performance yielded a relative standard deviation of <9%, *N* = 3, across the concentration range studied. The sensors’ responses are not linear over the entire concentration range but rather present saturation behavior at higher fentanyl concentrations. This suggests that the adsorption of fentanyl onto the fCNF/SPCE surface follows a Langmuir or bi-Langmuir model,^36,37^ which was confirmed by a good fit to the Langmuir isotherm equation, with an *R*^2^ of 0.9999. From the fit, the maximum oxidation current at saturation is 46 μA and the adsorption capacity of the electrode (i.e., dissociation constant, *K*_d_) is 31 μM. The fit implies that fentanyl adsorption sites on the fCNF are energetically equivalent.^38^ According to equilibrium adsorption kinetics, the low concentration range of the isotherm is predicted to be approximately linear, and a linear regression fit between 0.125 and 10 μM yielded a straight line (*R*^2^ = 0.9999); see the inset of Figure 5b. The LOD of the sensor was calculated to be 75 nM based on 3σ/slope, where σ was the standard deviation at 0.125 μM fentanyl. Though the LOD of our sensor is better than or comparable to literature-reported values, see Table 1, work is still required to further reduce the detection limit. As indicated in the Introduction section, the lethal concentration of fentanyl has been experimentally determined to range from 3 to 600 nM.^4−6^

**Table 1:** Comparison of the Performance of the fCNF/SPCE with the Contemporary Literature-Reported Fentanyl Electrochemical Sensors^a^

sensor	method	matrix	LOD (μM)	linear range (μM)	reference
Zn(II)-MOF/SPCE	DPV	0.1 M PB, pH 7	0.3	1–100	(32)
MWCNT and NiO nanodisks/PGE	DPV	0.1 M PB, pH 7	0.0067	0.01–800	(39)
carbon nano-onions/GCE	DPV	0.1 M PB, pH 7	0.3	1–60	(19)
SWCNTs/glass	DPV	PBS, pH 7.4	0.011	0.01–1	(20)
SPCE	SWAdSV	0.1 M Tris–HCl, pH 8.5	0.11 (m1), 0.69 (m2)	0.23–20.5	(24)
ionic liquid/SPCE	CSWV	0.1 M PB, pH 7.4	5	10–100	(33)
MWCNTs and ionic liquid/SPCE	SWV	0.1 M PB, pH 7.4	10	10–100	(22)
laser carbonized electrode	SWV	0.1 M PBS	1	20–200	(25)
MWCNT/GCE	DPAdSV	0.1 M PB, pH 7.4	0.1	0.5–100	(21)
fCNFs/SPCE	DPV	0.1 M PB, pH 8.0	0.075	0.125–10	this work

a MOF = metal–organic framework, PGE = pencil graphite electrode, GCE = glassy-carbon electrode, SWAdSV = square-wave adsorptive stripping voltammetry, CSWV = cyclic SWV, PVC = polyvinyl chloride, RGO = reduced graphene oxide, NPs = nanoparticles, CPE = carbon paste electrode, eRGO = electrochemically RGO, CP = carbon paste, PBS = PB saline, SWV = square-wave voltammetry, DPAdSV = differential pulse adsorptive stripping voltammetry. m1 measurement done in a 5 mL electrochemical cell and m2 measurement done using a drop (100 μL) of fentanyl.

### Long-Term Electrode Stability

The long-term stability of the fCNF/SPCEs was tested for several weeks. Eight electrodes were prepared in parallel and stored in a desiccator under vacuum at room temperature before use. The electrodes were tested against 10 and 20 μM fentanyl solutions prepared in 0.1 M PB pH 8.0. Figure S6 shows the OX1 peak currents for each electrode over 8 weeks. As shown in the figure, the average current values for the duration of the experiment were 14.4 ± 0.69 μA (% RSD = 4.8%) and 21.1 ± 0.83 μA (% RSD = 4.0%) for 10 and 20 μM fentanyl, respectively. A single fCNF/SPCE sensor was also tested against 5 μM fentanyl 5 times, yielding a % RSD of 6.7%. The data presented here attest to the reproducibility of the sensor preparation method and suggest that electrode storage is feasible for a minimum of 8 weeks.

### Selectivity of the Sensor

**Figure 6:**
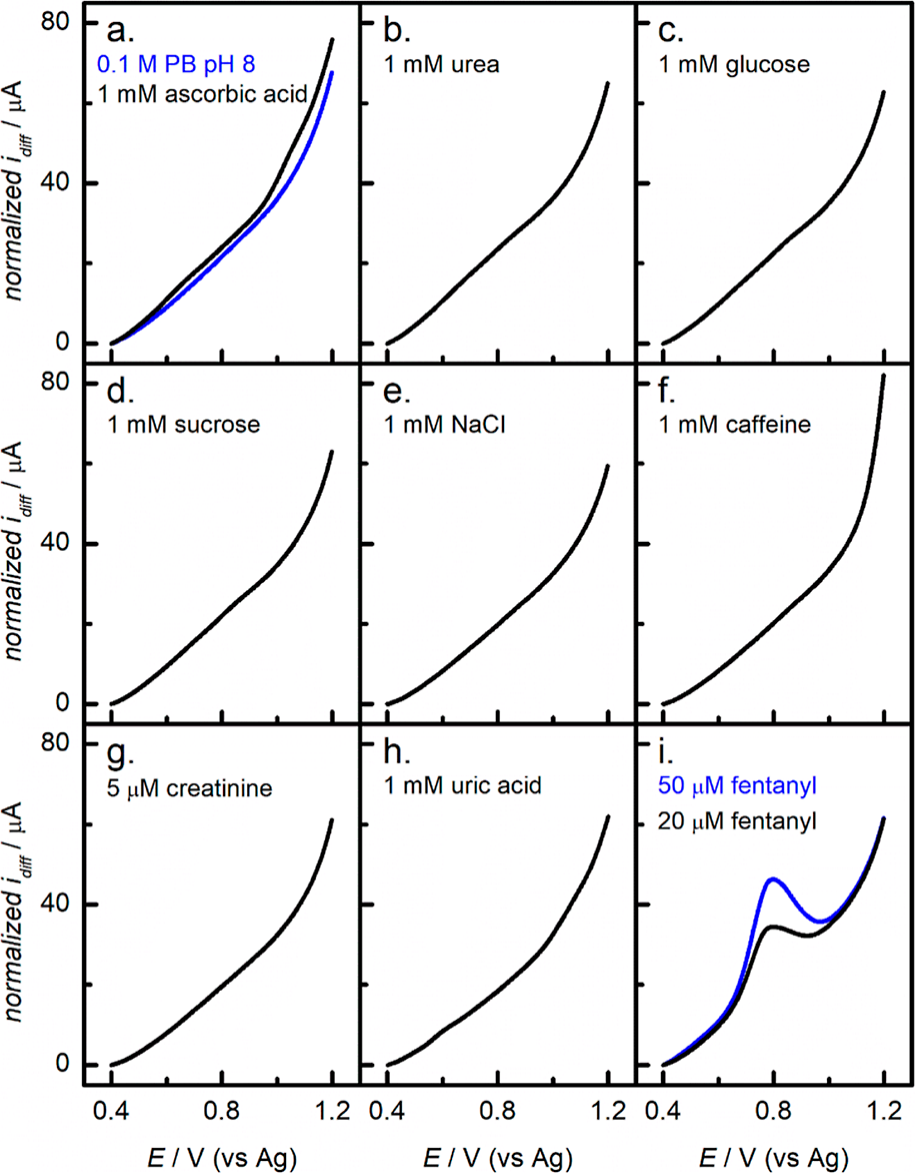
DPV voltammograms of possible fentanyl interferents: (a) 1 mM ascorbic acid, (b) 1 mM urea, (c) 1 mM glucose, (d) 1 mM sucrose, (e) 1 mM NaCl, (f) 1 mM caffeine, (g) 5 μM creatinine, (h) 1 mM uric acid, and (i) 20 and 50 μM fentanyl. Panel (a) also contains a voltammogram for the electrolyte, 0.1 M PB at pH 8, used for all measurements.

The selectivity of the fCNF/SPCEs toward fentanyl was first tested against common interfering compounds found in biofluids (i.e., urea, ascorbic acid, caffeine, NaCl, sucrose, glucose, creatinine, and uric acid prepared individually at 1 mM, except for creatinine which was prepared at 5 μM). For this assessment, a single fCNF/SPCE was utilized to acquire all measurements in the sequence shown in Figure 6 (i.e., from panel a–i) with aqueous rinsing and drying under N_2_ after each measurement. The final test consisted of repeating the measurement of fentanyl as a positive control to ensure electrode functionality. Despite the sensor showing no response to the studied interfering compounds, the signal for fentanyl measured at the end of the sequence decreased by 39% for 20 μM and by 28% for 50 μM when compared to the data presented in the calibration curve (Figure 5b). This suggests that the surface of the fCNF/SPCE sensor was either partially covered or poisoned by the multiple interferent measurements or some amount of fCNF was lost during the 8 rinsing and drying cycles. Nevertheless, the fact that the sensor responded to fentanyl, even after the extensive testing sequence, indicates its suitability for use in biofluids.

**Figure 7:**
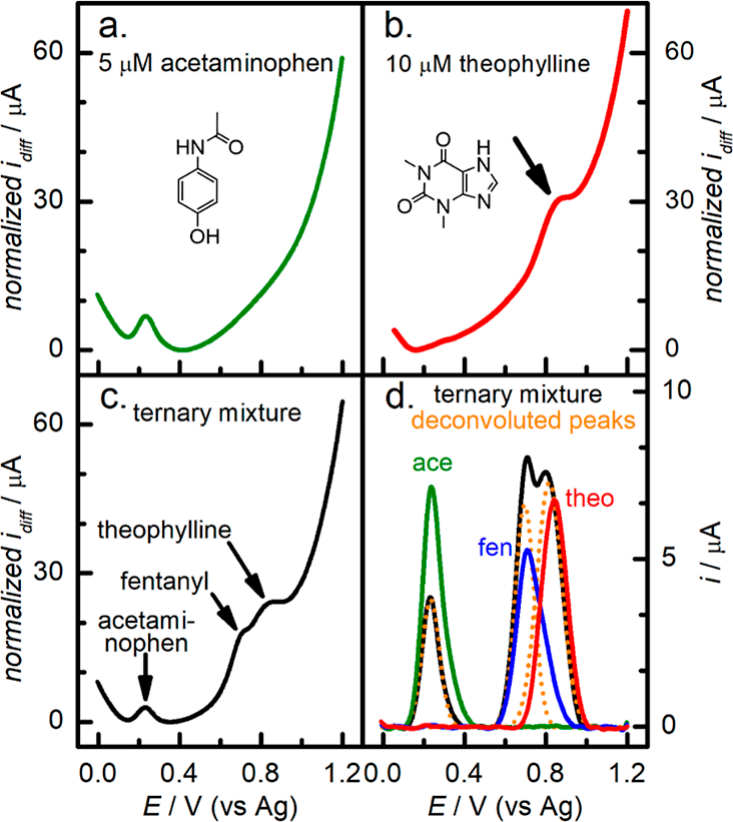
DPV voltammograms of (a) 5 μM acetaminophen, (b) 10 μM theophylline, a (c) ternary mixture containing 5 μM acetaminophen, 5 μM fentanyl, and 10 μM theophylline, and (d) baseline-subtracted voltammograms of acetaminophen (green trace), fentanyl (blue trace), theophylline (red trace), and the ternary mixture (black trace) along with its 3 deconvoluted peaks (orange dotted traces). All analytes were diluted in 0.1 M PB pH 8.0.

Glucose, sucrose, caffeine, acetaminophen, and theophylline are some of the common cutting agents found in street drugs.^22,33^ Since the sensor did not respond to glucose, sucrose, and caffeine, as shown in Figure 6, we investigated its response to acetaminophen and theophylline, first by monitoring the DPV response of the sensor to individually prepared 5 μM acetaminophen and 10 μM theophylline and then to a ternary mixture with 5 μM fentanyl. Fresh electrodes were utilized for each measurement to avoid the residual cross-contamination observed in the previous study. Figure 7a shows that the acetaminophen oxidation potential is much lower than that of fentanyl at 0.24 V (vs Ag). Conversely, the oxidation potential for theophylline, Figure 7b, shows a peak at 0.84 V (vs Ag) or about 0.12 V higher than fentanyl’s OX1 peak. A mixture of the three components presented in Figure 7c displays three peaks, indicating that the sensor can discriminate among these components. Although the fentanyl and theophylline peaks can be discriminated, the signals are not completely resolved, and the peak currents cannot be accurately measured without deconvolution. Upon implementing a deconvolution process (described in the Experimental section), a significant peak separation was observed and the peaks in the mixture are completely resolved, enabling reliable measurement of the individual analyte peak intensities and areas. Analyses of the mixture DPV voltammogram indicate that acetaminophen’s peak is reduced 55% by area and 47% by intensity when compared to its individual DPV signal. The deconvolution of theophylline’s peak shows an increase of 8% in both area and intensity when mixed, which is within the experimental error for multiple electrodes. The deconvoluted peak for fentanyl in the mixture shows a decrease of 11% in area and an increase of 28% in intensity. The reduced acetaminophen signal is most likely due to competition between the components in the mixture and to its lower favorable interaction with the surface. In general, for deconvoluted peaks, calculated peak area is usually more representative than peak intensity, with our mixture having an increased area of ∼10% for both theophylline and fentanyl.

### DPV in Artificial Urine as a Matrix

Urine testing has been widely used when assessing opioid consumption.^40−42^ Although ∼90% of fentanyl is excreted after metabolic breakdown, the original form is also detectable in urine for less than 72 h in medical patients^42^ and up to 4 weeks in regular users.^41^ Since the effects of fentanyl only last a few hours, rapid detection in biological samples, such as urine, is a useful approach for monitoring subjects.

**Figure 8:**
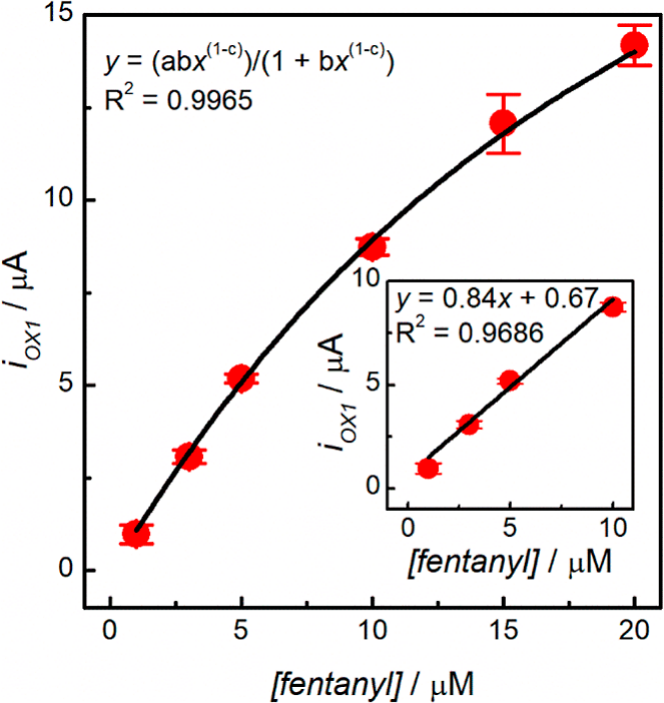
OX1 peak currents extracted from DPV voltammograms of different fentanyl concentrations in a 1:10 artificial urine/0.1 M PB pH 8.0 matrix. Data points represent the average currents extracted from 3 electrodes with a Langmuir fit (black trace). The fitted Langmuir equation constants are *a* = 30 μA, *b* = 38 × 10^–3^ μM^–1^, and *c* = −54 × 10^–3^. The inset is a zoomed-in section of the data set with fentanyl concentrations ≤10 μM with its independent linear fit.

The developed fCNF/SPCE sensor was tested for fentanyl determination in an artificial urine matrix (composition shown in Supporting Information Table S1). The artificial urine was diluted 1:10 in 0.1 M PB at pH 8, then fentanyl was spiked into the sample with varying concentrations, and the DPV signals were recorded. Figure 8 shows that the OX1 peak current follows the Langmuir isotherm (*R*^2^ = 0.9965) in the studied concentration range of 1–20 μM and displayed a linear relationship for 1–10 μM fentanyl (see inset of Figure 8), which is consistent with Figure 5b. Comparing the data in Figures 8 to 5b, the response of the sensor to fentanyl in urine samples was reduced to ∼58% of what was measured in the buffer, indicating that the constituents of the urine sample have an interfering effect, which is in accordance with the results presented in Figure 6 where partial fouling of the sensor was noted upon exposure of the sensor to individual urine components. Using the linear fit in the 1–10 μM fentanyl concentration range, we calculated an LOD of 0.9 μM fentanyl in the artificial urine matrix.

## Conclusions

We have demonstrated the use of COOH-functionalized CNFs on an SPCE as a suitable approach for the electrochemical detection of fentanyl. The CV profiles for the electro-oxidation of fentanyl showed two oxidation peaks (OX1 and OX2) associated with the dealkylation of the tertiary amine and two additional sets of redox peaks. The redox pair of OX3/R2 is associated with norfentanyl, the oxidation product of fentanyl, while the source of the previously uncatalogued OX4/R1 pair is currently unknown. Optimizing fCNF loading, pH of electrolyte solution, and fentanyl accumulation time resulted in a calculated fentanyl LOD of 75 nM in a 0.1 M PB pH 8 matrix via DPV. Artificial urine and several individual components showed no electrochemical signal interference under the optimized experimental conditions; however, electrode poisoning resulted in lower OX1 current signals. The sensor was also tested against common cutting agents found in street drugs and was found to be capable of discriminating fentanyl from a mixture with theophylline and acetaminophen. Finally, the sensor storage stability for an 8 week period indicated little deterioration, suggesting adequate shelf life and assembly procedures.

## Supporting Information Available

Additional experimental details characterizing the fCNFs via Raman spectroscopy, scanning electron microscopy, and electrochemical techniques, storage stability of the sensor, and the composition of artificial urine used (PDF)
