# Supplementary material for: Electrochemical Determination of Fentanyl Using Carbon Nanofiber-Modified Electrodes
Source: ACS Omega. 2024 Apr 5;9(15):17592–601. doi: 10.1021/acsomega.4c00816 (PMC11024940; doi:10.1021/acsomega.4c00816)
Supplement: Supplementary file 1 — ao4c00816_si_001.pdf [file ao4c00816_si_001.pdf]

Supporting Information

Electrochemical determination of fentanyl using carbon nanofiber-modified electrodes

Armando J. Marenco[1], Rajesh G. Pillai[1], Kenneth D. Harris[1,2], Nora W.C. Chan[3*], and Abebaw B. Jemere[1,4*]

[1] National Research Council Canada – Nanotechnology Research Centre, 11421 Saskatchewan Drive, Edmonton, AB T6G 2M9, Canada
[2] Department of Mechanical Engineering, University of Alberta, Edmonton AB T6G 1H9, Canada
[3] Defence Research and Development Canada, Suffield Research Centre, P.O. Box 4000 Stn. Main, Medicine Hat, AB T1A 8K6, Canada
[4] Department of Chemistry, Queen's University, Kingston, ON, K7L 3N6

[*] Corresponding authors: nora.chan@forces.gc.ca; abebaw.jemere@nrc-cnrc.gc.ca

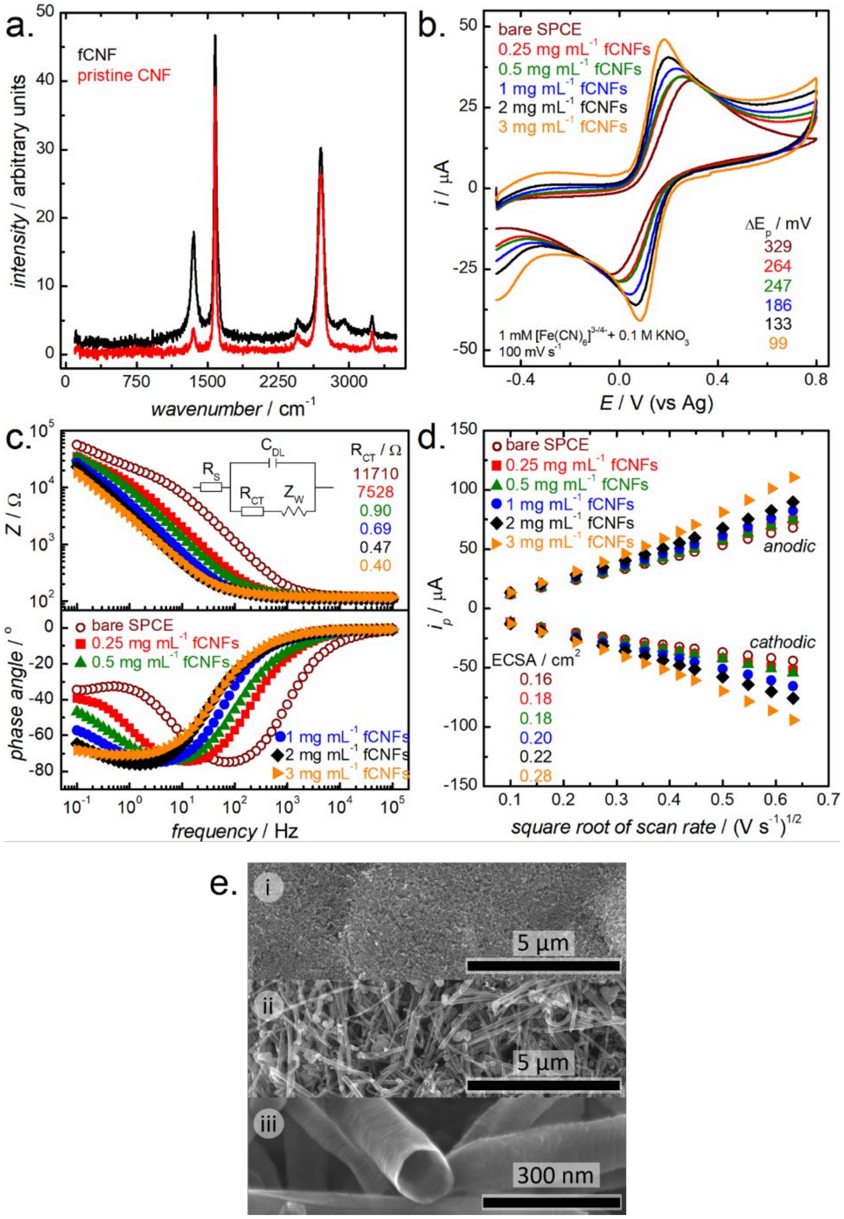

Figure S1. Characterization of unmodified and fCNF-modified SPCEs. (a) Raman spectra of pristine and acid functionalized carbon nanofiber, (b) sample CVs for a bare SPCE and fCNF/SPCE electrodes prepared with 0.25, 0.5, 1, 2, and 3 mg mL^{-1} fCNFs in 1 mM $[Fe(CN_6)]^{3-/4-}$, and their corresponding ΔE_p. (c) The Bode plots from EIS measurements of the electrodes presented in (b), the Randles circuit fit, and corresponding best-fit R_{CT} values. (d) The anodic and cathodic peak currents of the electrodes presented in (a) at scan rates ranging from 10 to 400 mV s^{-1}, and corresponding ECSAs. (e) The SEM images of (i) a bare SPCE electrode, (ii) a 1 mg/mL fCNF-modified SPCE, and (iii) the expanded view of a fCNF showing its hollow core.

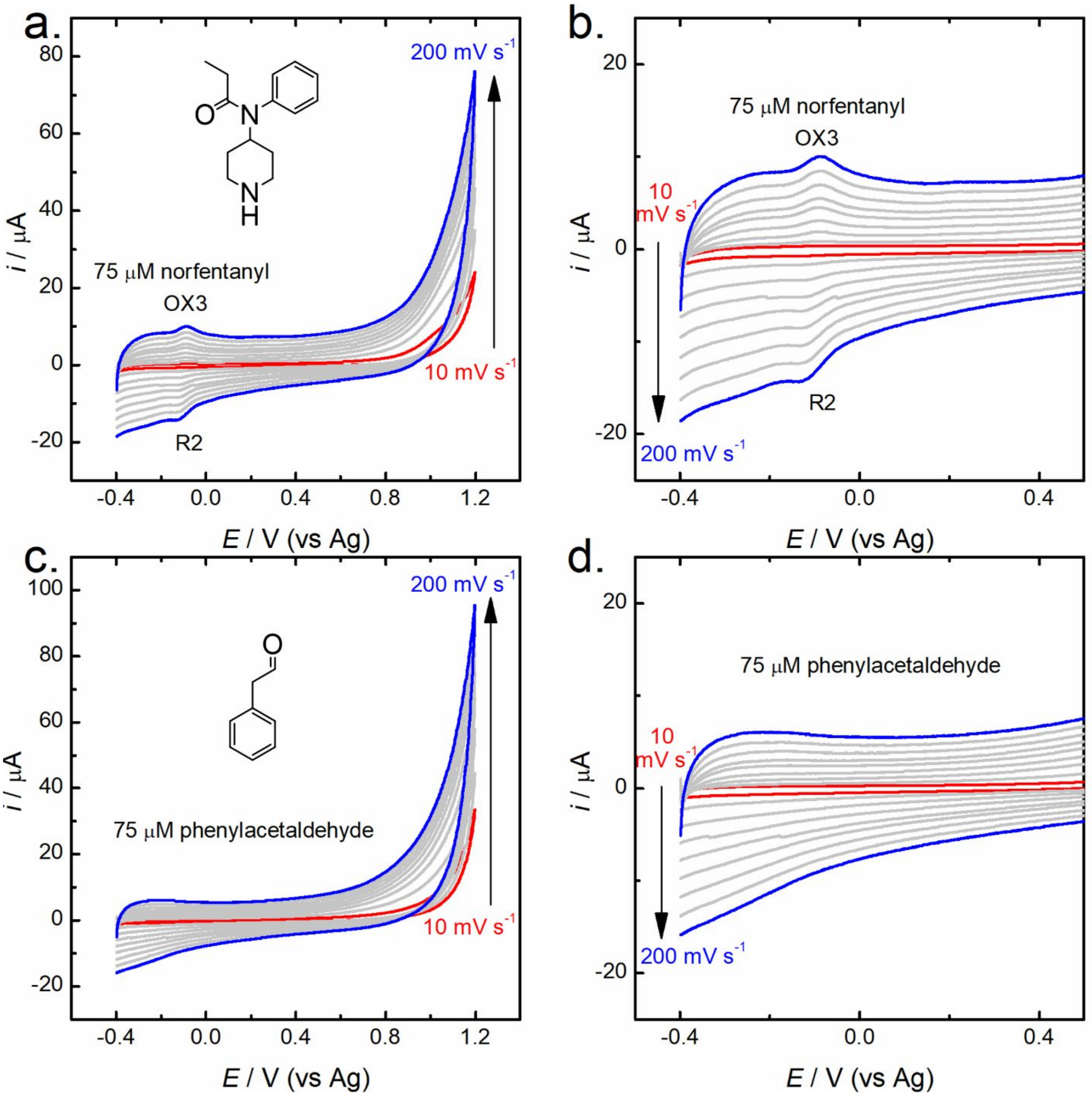

Figure S2. Cyclic voltammograms of (a and b) norfentanyl and (c and d) phenylacetaldehyde with an SPCE electrode modified with a 1 mg mL^{-1} fCNFsuspension. (a) A 75 µM norfentanyl solution tested at 10-200 mV s^{-1} scan rates between -0.4-1.2 V. (b) A close up of (a) showing the OX3 and R2 redox peaks. (c) A 75 µM phenylacetaldehyde solution tested at 10-200 mV s^{-1} scan rates between -0.4-1.2 V. (d) A close up of (c) showing the absence of peaks. The electrolyte in all cases was 0.1 M PB pH 8.0 buffer.

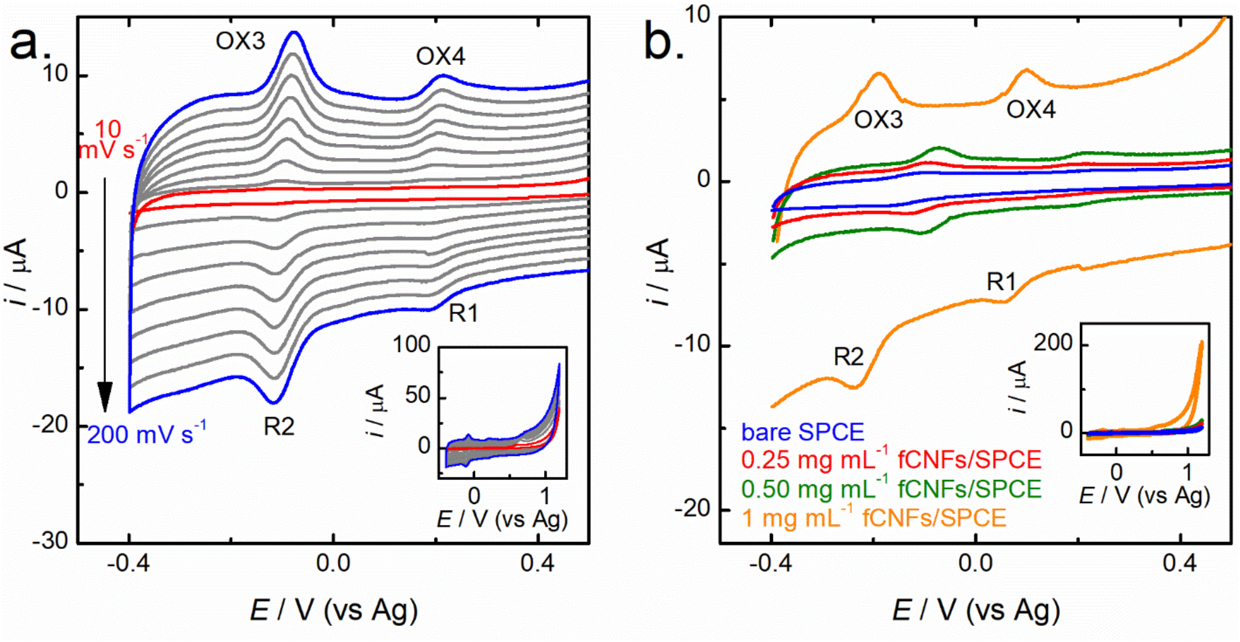

Figure S3. Cyclic voltammograms of several SPCE electrodes in the presence of 75 µM fentanyl showing the R1/OX4 and R2/OX3 redox peaks. (a) 2 mg/mL fCNFs/SPCE at different scan rates ranging from 10 to 200 mV s^{-1}. (b) SCPEs modified with 0, 0.25, 0.50 and 1 mg mL^{-1} fCNFs showing the 5th cycle at 100 mV s^{-1}.

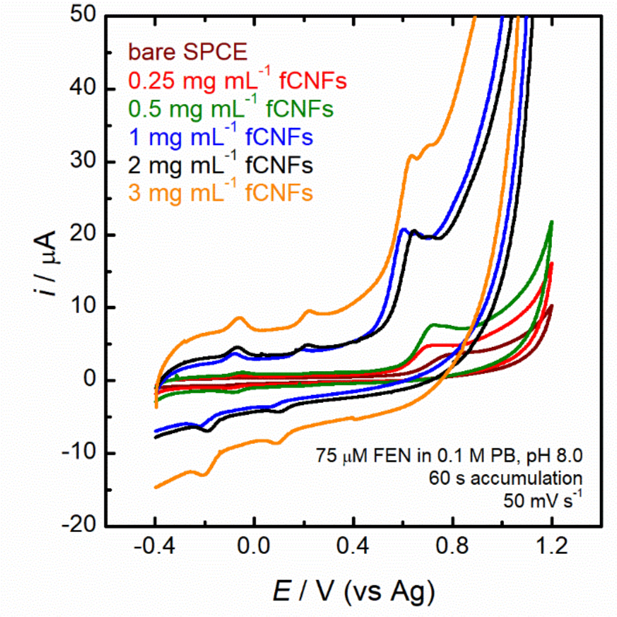

Figure S4. Cyclic voltammograms of several SPCE electrodes in the presence of 75 µM fentanyl after 60 s accumulation time. SCPEs modified with 0, 0.25, 0.50, 1, 2 and 3 mg mL^{-1} fCNFs. CVs acquired at 50 mV s^{-1}.

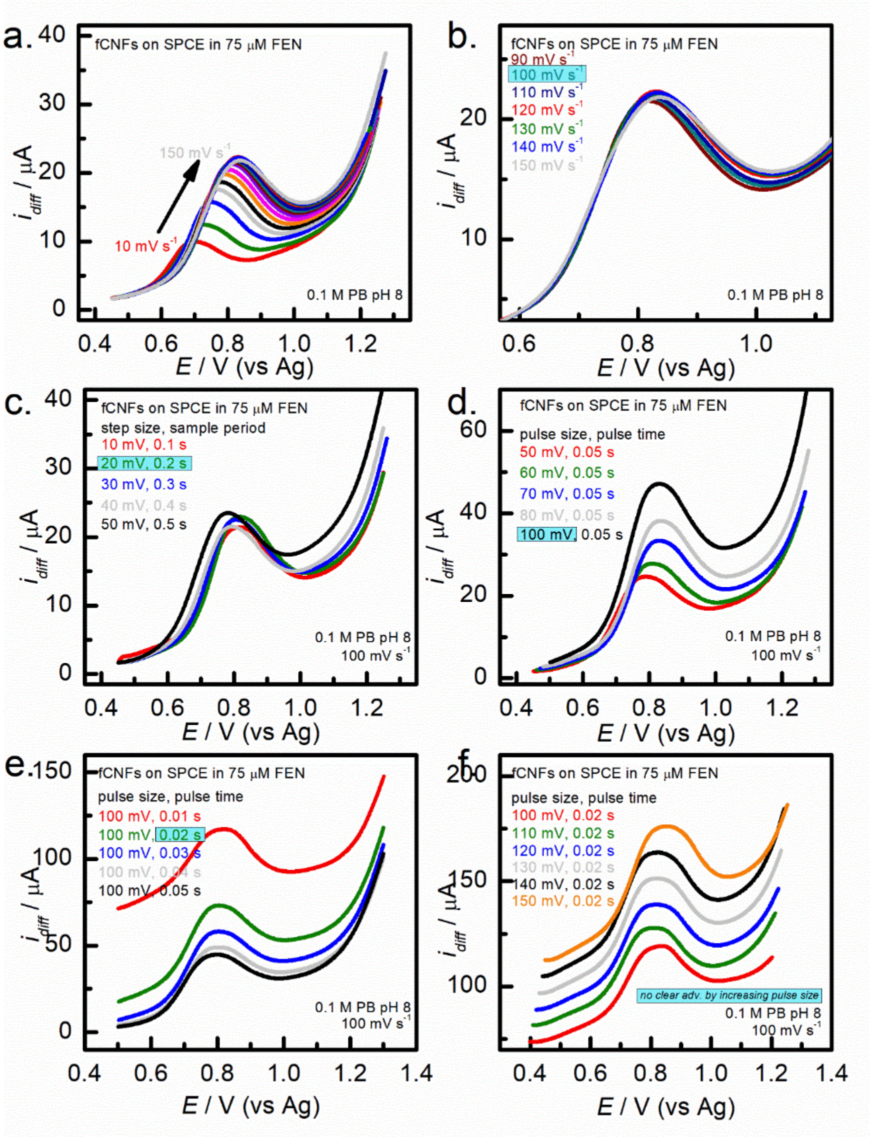

Figure S5. DPV parameter optimization. (a) Scan rates from 10 to 150 mV/s in increments of 10 mV/s. (b) Close up of selected scan rates. 100 mV/s was selected as optimal. (c) Step size and sample period optimization for scan rates of 100 mV/s. 20 mV step size and 0.2 s sample period were selected as optimal. (d) Pulse size optimization while keeping the pulse time constant. 100 mV pulse size was selected as optimal. (e) Pulse time optimization while keeping the pulse size constant. 0.02 s was selected as optimal. (f) Testing the effect of pulse size on the pulse time. No clear advantage was observed by increasing pulse size, and thus optimized parameters were based on the data in (d) and (e).

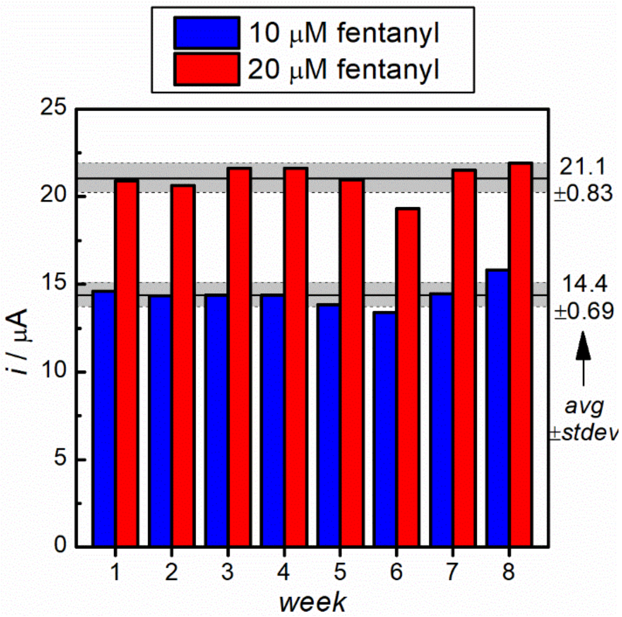

Figure S6. The OX1 current responses via DPV of several 1 mg/mL fCNF-modified SPCE electrodes exposed to 10 μM and 20 μM fentanyl during a single-use weekly study. All electrodes were prepared during week 1 and stored under vacuum, while retrieving 1 modified electrode per week for these studies. The gray horizontal lines indicate the average currents, while the dotted lines show the standard deviation values.

Table S1. Composition of artificial urine.

Component	Composition (%)
Water, distilled water, deionized water	90-99
Urea	≤ 2.5
Creatinine hydrochloride	< 0.5
Sodium chloride	< 1.0
Sodium phosphate, dibasic	< 0.5
Potassium phosphate	< 0.5
Sodium sulphite	< 0.5
Ammonium chloride	< 0.5
